# Extracellular Cues Govern Shape and Cytoskeletal Organization in Giant Unilamellar Lipid Vesicles

**DOI:** 10.1021/acssynbio.2c00516

**Published:** 2023-01-18

**Authors:** Andreas Fink, Charlotte R. Doll, Ana Yagüe Relimpio, Yannik Dreher, Joachim P. Spatz, Kerstin Göpfrich, Elisabetta Ada Cavalcanti-Adam

**Affiliations:** † Department of Cellular Biophysics, Max Planck Institute for Medical Research, Jahnstraße 29, 69120 Heidelberg, Germany; ‡ Institute for Molecular Systems Engineering, University of Heidelberg, Im Neuenheimer Feld 253, 69120 Heidelberg, Germany; § Biophysical Engineering Group, Max Planck Institute for Medical Research, Jahnstraße 29, 69120 Heidelberg, Germany; ∥ Department of Physics and Astronomy, Heidelberg University, 69120 Heidelberg, Germany

## Abstract

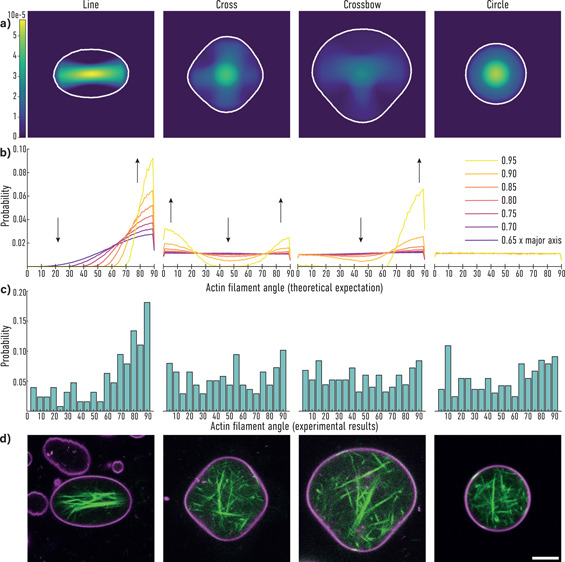

Spontaneous and induced front-rear polarization and a subsequent asymmetric actin cytoskeleton is a crucial event leading to cell migration, a key process involved in a variety of physiological and pathological conditions such as tissue development, wound healing, and cancer. Migration of adherent cells relies on the balance between adhesion to the underlying matrix and cytoskeleton-driven front protrusion and rear retraction. A current challenge is to uncouple the effect of adhesion and shape from the contribution of the cytoskeleton in regulating the onset of front-rear polarization. Here, we present a minimal model system that introduces an asymmetric actin cytoskeleton in synthetic cells, which are resembled by giant unilamellar lipid vesicles (GUVs) adhering onto symmetric and asymmetric micropatterned surfaces. Surface micropatterning of streptavidin-coated regions with varying adhesion shape and area was achieved by maskless UV photopatterning. To further study the effects of GUV shape on the cytoskeletal organization, actin filaments were polymerized together with bundling proteins inside the GUVs. The micropatterns induce synthetic cell deformation upon adhesion to the surface, with the cell shape adapting to the pattern shape and size. As expected, asymmetric patterns induce an asymmetric deformation in adherent synthetic cells. Actin filaments orient along the long axis of the deformed GUV, when having a length similar to the size of the major axis, whereas short filaments exhibit random orientation. With this bottom-up approach we have laid the first steps to identify the relationship between cell front-rear polarization and cytoskeleton organization in the future. Such a minimal system will allow us to further study the major components needed to create a polarized cytoskeleton at the onset of migration.

## Introduction

Polarity is found in biological systems at many different scales, ranging from molecules to cells and embryos, and it is known to be prerequisite for actin cytoskeleton organization and actin-driven cell motility. While extracellular cues, e.g., gradients and confinement, and the resulting molecular organization and dynamics driving front-rear polarity have been extensively identified, what remains to be determined is the interplay between actin organization and the imposed changes in cell shape. In a cell, such distinct regulation is difficult to identify, due to the presence of signaling events, which coparticipate in the regulation of cell structure dynamics.^1^ Cytoskeletal symmetry breaking is key for the formation of protrusions in cell movement, and it depends on cell membrane properties, including structure, organization, and shape.^2^ A remarkable example is the symmetry break found in adhesive migrating cells like keratocytes and, more interestingly, fragments of their lamellipodia. When adhered, but not motile, the fragments are generally circular, with a nonpolarized cytoskeleton. Moving fragments, on the other hand, show a highly polarized cytoskeleton, kept in a stable dynamic state by actin retrograde flow and myosin contraction.^3^ A recent study has identified an actin branched network against the membrane as the main acting element for the maintenance of protrusions such as lamellipodia.^4^ To gain better control over such polarized systems in vitro, surface micropatterning has been used to study the relation between shape, function, and polarity in a more stationary approach as a function of cell-extracellular surface adhesion.^5,6^ Using asymmetric, adhesive micropatterns like crossbow shapes, single cells are polarized in a reproducible manner; this control of cell shape is largely independent of cell type.^7−10^ To implement the extracellular control of an asymmetric cytoskeleton in a bottom-up approach, in this work we present a minimal system for deforming adherent giant unilamellar lipid vesicles (GUVs). Different systems for deforming GUVs, from the outside and inside, have been used in the past, such as DNA origami or microfluidic devices.^11−14^

Here we use surface micropatterning by maskless UV-photolithography to generate adhesive regions functionalized with streptavidin and surrounded by passivated regions on the surface. Although similar setups have been used in the past to to study membrane fluctuations in adhered GUVs, in the present work focus is laid on the deformation of biotin-functionalized GUVs that adhere to symmetric and asymmetric patterns of different shape and size.^15^ We further incorporate actin filaments in the GUVs to create a minimal synthetic cell suitable to study the distinct relationships between cell shape and actin cytoskeleton organization.

## Results and Discussion

**Figure 1:**
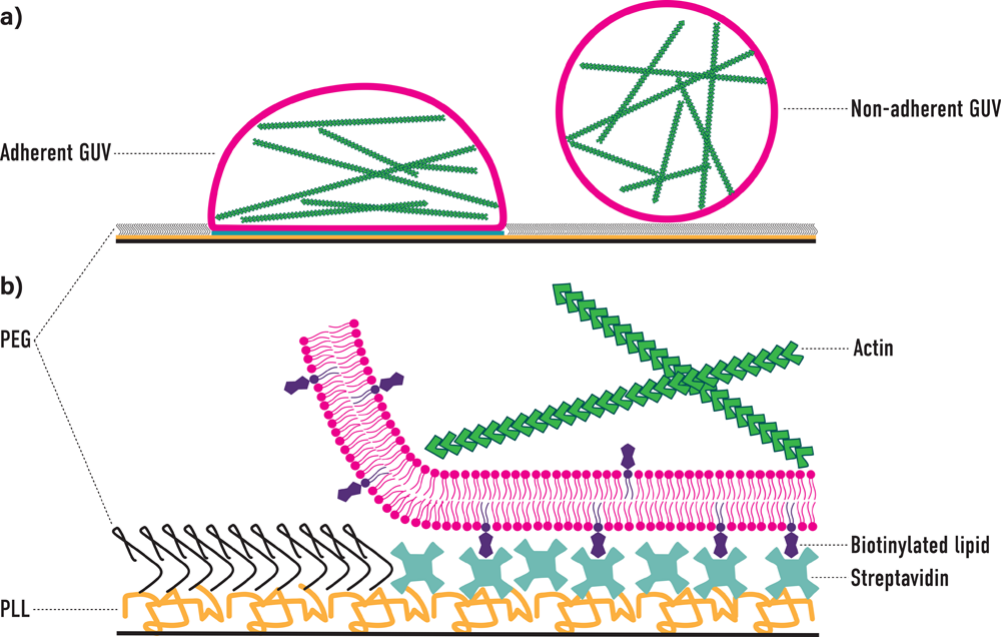
Schematic illustration of actin-containing GUVs on a micropatterned surface. (a) Sketch of an adherent (left) and a nonadherent actin-filled GUV (right). (b) Detailed view of the adhesion area. GUVs containing biotinylated lipids can only adhere where the PEG has been removed during the micropatterning process and was backfilled with streptavidin.

First, we set out to achieve controlled and site-specific adhesion of GUVs onto patterned surfaces to induce defined vesicle shapes (see Figure 1a). For this purpose, GUVs were engineered to adhere to micropatterend surfaces (see Figure 1). To this end, we supplemented a lipid mixture containing DOPC (1,2-dioleoyl-*sn*-glycero-3-phosphocholine) and DOPG (1,2-dioleoyl-*sn*-glycero-3-phospho-(1′-rac-glycerol) (sodium salt)) with 1% biotinylated lipids to adhere the GUVs to streptavidin-coated micropatterns. These were created by first coating coverslips with mPEG-SVA (methoxypoly(ethylene glycol) succinimidyl valerate) and micropatterning them using maskless photolithography, degrading the PEG layer in the illuminated areas. The regions were then filled with fluorescently labeled streptavidin, allowing us to directly visualize the created patterns. The biotinylated GUVs adhere to the patterns, as illustrated in Figure 1a,b. In some experiments, actin monomers were added to the inside of the GUVs to study the effect of deformation on actin filament organization.

**Figure 2:**
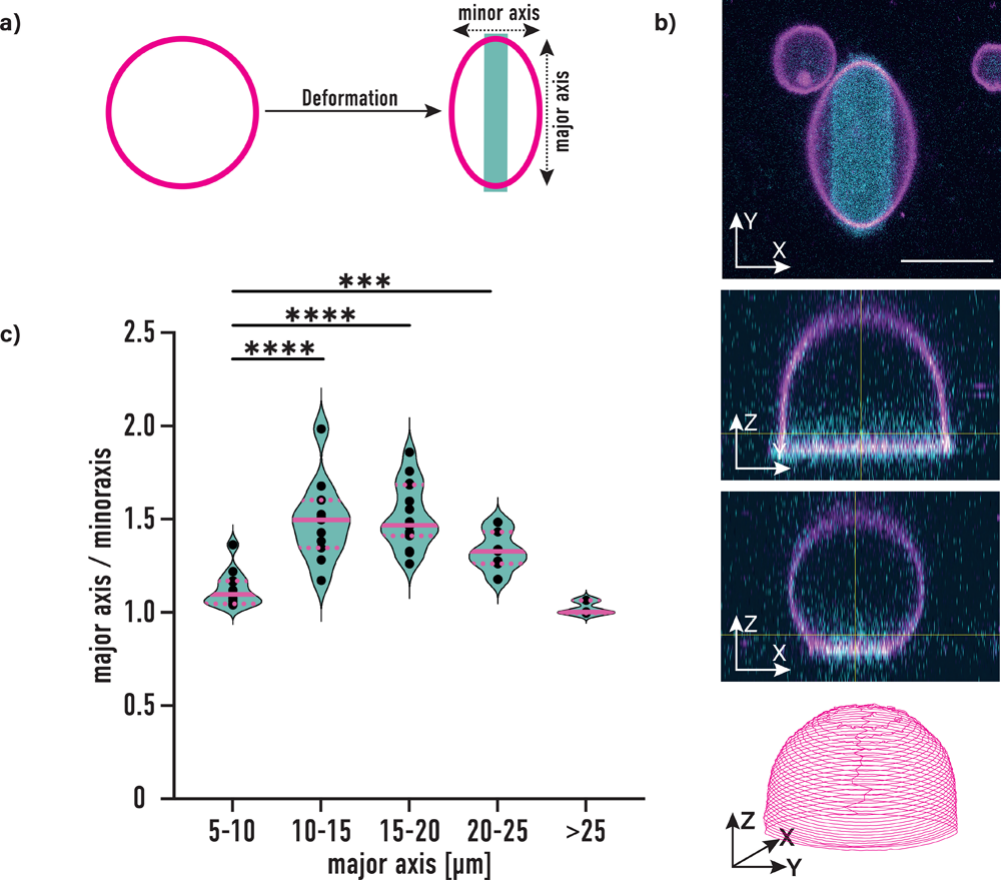
Analysis of GUV deformation on 15 μm long stripe micropatterns. (a) Sketch (top view) showing the expected deformation of an initially spherical GUVs adhering to a stripe micropattern. (b) Representative image of a deformed GUV (magenta) on a stripe pattern (cyan). The xy as well as yz (along the stripe) and xz (cut through the stripe) projections are shown. A 3D reconstruction for better visualization is shown. The scale bar is 10 μm. (c) Analysis of GUV deformation in dependence of GUV size. GUVs with a size comparable to the stripe length show the strongest deformation. A representative image for each group is shown in the Supporting Information, Figures S2–S6. The group of 5–10 μm GUVs has a sample size of 11, 10–15 μm GUVs of 11, 15–20 μm GUVs of 15, 20–25 μm GUVs of 7, and >25 μm GUVs of 3. 47 GUVs from four independent experiments analyzed in total; violin plots show the mean value (magenta line), **** *p* < 0.0001 and *** *p* < 0.001.

GUV deformation was analyzed in a quantitative manner for stripe patterns having 15 μm length. While a line width of 2 μm was used in the photomask, the obtained line width is in the range of 5 μm, and it varies between samples due to defocusing during the patterning process (see Figure S1). In Figure 2a the expected deformation of a GUV adhering to a stripe pattern is depicted together with the definition for the minor and major axis. A representative confocal microscopy image 2 μm above the surface (defined as the z-plane with the highest intensity of the Streptavidin-Alexafluor405 channel), as well as xz and yz-cut of an adherent and deformed GUV, are shown in Figure 2b. Further, the three-dimensional (3D) shape was reconstructed from z-stacks. GUV deformation is substantial above the pattern, and a more spherical shape at the top further away from the adhesion pattern is evident.

The ratio between the major and minor axes of deformed GUVs was analyzed and compared in order to quantify their deformation (Figure 2c). The membrane shape at the image plane 2 μm above the micropattern was used to quantify the deformation. Here GUV size affects their deformation: for small GUVs, with sizes comparable to the pattern width, the ratio of major and minor axes is close to 1, which corresponds to a circular shape. The GUVs cannot adapt to the asymmetric shape of the pattern, as their adhesion area is not limited in any axes, and therefore it is fully symmetric. For GUVs that are larger than the pattern width, but not much larger than its length, significant deformation of the GUVs is expected, as their adhesion is limited by the pattern width but not its length. For large GUVs with sizes much larger than the pattern length, the effect is expected to decrease, as the induced deformation is small in comparison to the overall size. The GUVs take on a circular shape closely above the pattern again.

**Figure 3:**
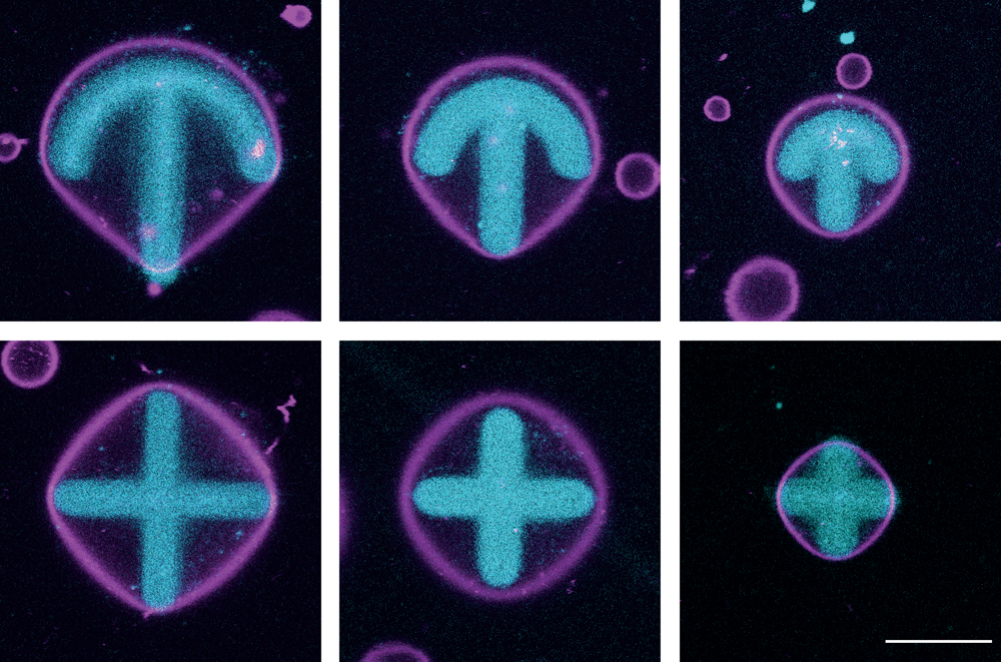
Representative confocal microscopy images of GUVs (magenta) adhering onto 20, 15, and 10 μm wide crossbow and cross micropatterns (cyan). The scale bar is 10 μm. The membrane in the images is shown 2 μm above the pattern.

The wider applicability of using micropatterning to deform GUVs was investigated by adhering them to patterns of different sizes and shapes. Since crossbow patterns are widely used with cells to impose front-rear polarity, we used this type of pattern and compared GUV deformation to the one induced by patterns with 10, 15, and 20 μm in diameter. Representative confocal microscopy images of adherent, deformed GUVs 2 μm above the surface are shown in Figure 3. Here, the pattern is shown directly on the surface, and it is evident that GUVs of different size adhere on such patterns. Thus, surface micropatterning techniques can be widely applied to study adhesion-mediated GUV deformation.

**Figure 4:**
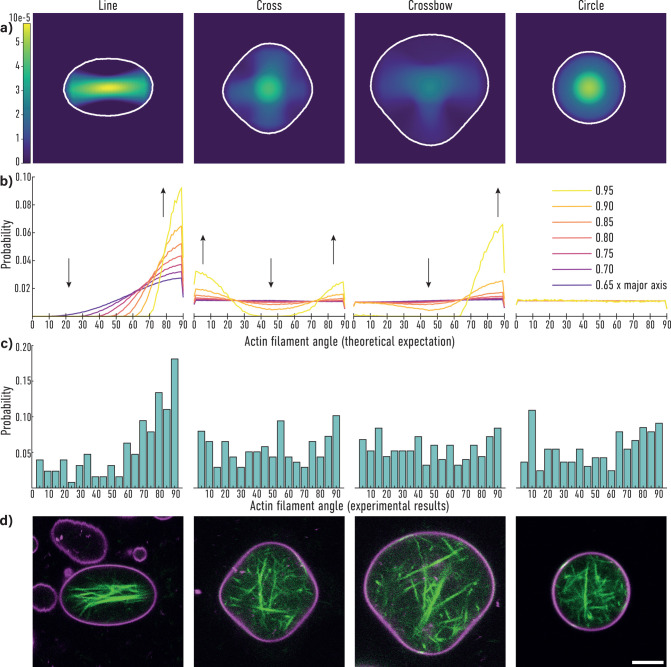
(a) Calculated heat maps depicting the actin probability density, normalized to 1 for the whole image for filaments with a length of 0.90× major axis. (b) Angle probability distributions extracted from generated heat maps with filament lengths in units of the length of the major axis of the depicted GUV. The arrows indicate changes in the distributions with increasing filament length. (c) Angle probability distributions extracted from experimental data. For the distributions on the line-shaped micropattern, 17 GUVs were used; for the cross and crossbow patterns, 12 and 13 GUVs were analyzed, respectively. For the distribution on circular pattern, 6 GUVs were used. (d) Microscopy images of actin containing GUVs 2 μm above micropattern. The scale bar is 5 μm.

Further, to show the applicability of micropatterns to deform GUVs, we studied the effect of the GUV shape on the organization of cytoskeletal proteins. To this end we used a theoretical and an experimental approach to determine the cytoskeletal orientation, shown in Figure 4. First, we computed all possible orientations and positions of filaments within a confined two-dimensional (2D) shape, resembling the adherent GUV. For this we extracted the GUV shapes from microscopy images and analyzed these further using Python.^16−19^ The scripts used to generate the data are given with a minimal working example in the Supporting Information. Assuming a rigid boundary, one can then calculate the possible orientations of actin filaments with a certain length for any point in the shape. For comparability the filament length is given in units of the length of the major axis. This allows for the generation of heat maps and angular distribution probabilities for actin filaments in deformed GUVs, assuming the actin filaments as straight lines, and their distribution to be only governed by the confinement shape. The results of this theoretical model are shown in Figure 4a,b. The outlines of the shapes used to calculate the distributions are shown in Figure 4a as the white contour lines and are taken from the microscopy images in Figure 4d, as described above, while the experimentally observed distributions are plotted in Figure 4c. The heat maps were computed for filament lengths ranging from 5 to 95% of the major axis of the corresponding GUV. Figure 4a shows the heat map corresponding to a length of 90% of the major axis. It can be seen that most filaments align along the major axis of the pattern.

For the analysis, the angles were mapped to [0, 90] degrees, assuming mirror symmetry between the left- and right-hand side. Figure 4b shows the expected angular distributions for different filament lengths for the shape seen in the heat map. The orientation of filaments that are short compared to the GUV size is random within the confinement, and therefore the distribution of these filaments is homogeneous. As the filaments get longer, this symmetry is broken for all shapes except for the circle. From the theoretical results with the cross and crossbow shapes it can be concluded that, for these shapes, the filaments have to have a length close to the size of the major axis to show asymmetric behavior in the angular distributions. Evidence for this behavior can be found in the steep change when the filament length goes toward the length of the major axis.

To verify the theoretical results, experimental data of actin containing GUVs adhering onto different shapes (see Figure 4c,d) were analyzed. For these experiments, actin was encapsulated together with the bundling protein fascin inside GUVs adhering on a 15 μm wide pattern. For the analysis of the filaments, the image plane 2 μm above the micropattern was chosen, as before. To extract the filament positions and orientation from the microscopy images, the open-source program SOAX^20^ was used, which traces the filaments and allows for further analysis. Using principle component analysis, the traced filaments were transformed into straight lines, and the rotational angle in 2D was calculated (see the Supporting Information for an example code).

When comparing the theoretical and experimental angle probabilities for the line micropattern in Figure 4b,c, a similar behavior, and thus, good agreement between theory and the experiment, can be seen. For the cross and crossbow micropattern, however, the experimentally obtained distributions show no asymmetry. This is explained well when considering that only filaments with a length close to the length of the major axis show an asymmetry in their angular distributions. In experimental GUVs, the majority of filaments appear to be shorter and, thus, show no strong asymmetry in the distribution. Indeed, the shape of such deformed GUVs resembles a circular GUV much more closely. This also becomes clear when looking at the theoretical distributions in Figure 4b. For GUVs on cross and crossbow micropatterns an actin filament needs to be approximately 90% of the GUV diameter or longer in order to be affected by the membrane geometry. Representative microscopy images of adhering GUVs with actin filaments are shown in Figure 4d. These GUVs were also used for the theoretical analysis in Figure 4a,b.

In summary, we present a method to deform adherent GUVs using micropatterning. Our approach allows for the reliable deformation of cell-sized compartments without the necessity of more complex microfluidic setups or external confinement by 3D micropatterning. Further, it widens the range of shapes GUVs can be deformed into, when compared to microfluidics. Moreover, such deformations induced by adhesion to the patterned substrates are comparable to those experienced by cells placed on similar patterned shapes.^7^ We see it therefore as a valuable tool to study adhesion, deformation, and with this the onset of polarization, as well as the geometrical implications, in a controlled manner.

For actin-containing GUVs, good agreement with the theoretical results could be obtained for GUVs deformed on line-shaped micropatterns. Actin filaments orientate along the major axis as expected. For GUVs adhering to cross and crossbow micropatterns, no preference of actin orientation can be observed in the experimental distributions. This can be explained when the theoretical results are taken into account, as actin filaments shorter than 0.85× the major axis length show an almost uniform distribution for all but line-shaped patterns. Additionally one can observe a variable strength in GUV deformation, depending on GUV size and the excess membrane area due to osmotic deflation. As shown in Figure 4, the shape of GUVs on these patterns is much closer to a circular one than on the line pattern. Furthermore, the theoretical investigations show that strong dependency of actin filament orientation on the GUV shape can only be observed for actin filaments with lengths similar to the length of the major axis.

## Materials and Methods

### GUV Production

All GUVs were produced using the emulsion transfer (also called inverted emulsion) method.^11,21^ The lipid-in-oil solution was prepared using a lipid mixture of 78.5% 1,2-dioleoyl-*sn*-glycero-3-phosphocholine (DOPC), 20% 1,2-dioleoyl-*sn*-glycero-3-phospho-(1′-*rac*-glycerol) (sodium salt) (DOPG), 1% 1,2-distearoyl-*sn*-glycero-3-phosphoethanolamine-*N*-[biotinyl (poly(ethylene glycol))-2000] (ammonium salt) (DSPE-PEG2K-Biotin), and 0.5% Atto 488 1,2-dioleoyl-*sn*-glycero-3-phosphoethanolamin (Atto488-DOPE) to achieve a final concentration of 643 μM in 1 mL of mineral oil. The lipids, dissolved in chloroform, were put in vacuum in a low-binding glass vial for 20 min to remove the solvent. The dried lipid film was then redissolved in 60 μL of *n*-decane. 940 μL of mineral oil was added and vortexed. Then 400 μL of this solution was added above 500 μL outside aqueous solution (5 mM imidazole-HCl buffer, pH 7.6, sucrose was added to match the osmolarity of the inside solution, here 305 mOsm) in a 2 mL Eppendorf tube. After an hour the lipids have formed a monolayer at the interface. Ten microliters of inside aqueous solution (for experiments without actin: A final concentration of 20% 5-[acetyl-[3-[acetyl-[3,5-bis(2,3-dihydroxypropylcarbamoyl)-2,4,6-triiodo-phenyl]amino]-2-hydroxy-propyl]amino]-*N*,*N*′-bis(2,3-dihydroxypropyl)-2,4,6-triiodo-benzene-1,3-dicarboxamide (Opti-prep) in phosphate-buffered saline (PBS); with actin, the final concentrations are given: ethylene glycol-bis(β-aminoethyl ether)-*N*,*N*,*N*′,*N*′-tetraacetic acid (EGTA), 1 mM, 15% OptiPrep, 5 μM imidazole-HCl buffer, pH 7.6, 2 mM Tris pH 8.0, 50 mM, 2 mM, 1 mM ATP), fascin (Hypermol EK, 0.5 μM), and 5 μM actin (purified after a protocol by Pradee and Spudich, modified by S.J. Kron et al.^22^) was added to the remaining lipid-in-oil solution. The mixture was pipetted up and down vigorously to create a water-in-oil emulsion. The emulsion was then carefully added on top of the oil layer on the outside buffer and centrifuged for 3 min at 380 rcf. Finally the oil residue on top was removed using a blunt end pipet tip in order to avoid GUV rupture due to shear flow. GUVs were prepared and used on the same day. To observe the actin filaments, 0.25 μL of SIR-actin stain was added to outside buffer of the GUVs containing actin.

### Micropatterning

Glass coverslips (18 × 18 mm) were treated with plasma at 200 W at 0.5 mbar for 3 min using a TePla 300 plasma machine. Then 200 μL of Poly-l-Lysine solution (0.01%, Sigma-Aldrich) was added onto each surface. After 30 min the coverslips were washed twice with HEPES, 10 mM, pH 8.0. Next, 5 mg of methoxypoly(ethylene glycol) succinimidyl valerate (MW 5000), dissolved in 100 μL HEPES, 10 mM, pH 8.0, was added onto each coverslip and left to incubate for 1 h at RT. The coverslips were washed with purified water, and 3 μL of 4-benzoylbenzyl-trimethylammonium chloride (PLPP-gel, Alveole) was added in 60 μL of ethanol (99.8%). After the surface dried, maskless photopatterning (Alveole PRIMO on Nikon inverted microscope) was used to generate the micropatterns. The surfaces were washed with PBS and stored in PBS in the fridge for up to one month. Before usage the coverslips were gently dried by letting the liquid run down the side onto a Kimtech wipe. Two microliters of Alexa-Fluor405 tagged streptavidin (Fischer Scientific, 2 mg/mL) was added in 100 μL of PBS and left to incubate on the surface for 1 h. Finally the solution was aspirated, and the surfaces were washed with 100 μL of PBS. Then 100 μL of the GUV suspension was added and imaged using an inverted confocal microscope (Zeiss LSM 880 Axio-Observer, equipped with a Plan-Apochromat 63*x*/1.4 Oil immersion objective). Atto4888-DOPE, *n*-Decane, Optiprep, and Mineral Oil was purchased from Sigma-Aldrich, Co. All other lipids were bought from Avanti Polar Lipids, Inc.

### Data Analysis and Presentation

All microscopy images shown in this work were created using Fiji/ImageJ.^23^ For the 3D representation of an adherent and deformed GUV in Figure 2b, a Python script was used to detect the GUV outline in each slice of the z-stack and plotted in 3D using gnuplot.^24^ For Figure 2c an unpaired *t* test was performed using Graphpad Prism between columns 1 (major axis 5–10 μm) and 2 (major axis 10–15 μm) and between columns 1 and 3 (major axis 15–20 μm) and columns 1 and 4 (major axis 20–25 μm), giving a two-tailed *p*-value of less than 0.0001, less than 0.0001, and 0.0008, respectively. For the heatmaps in Figure 4, Matplotlib 3.5 was used. The script for the heatmap generation is given in the Supporting Information. The probability distributions were plotted using Gnuplot. To calculate the actin orientation SOAX was used. To this end, first a Gaussian blur is applied to the microscopy image. Then the SOAX algorithm was run on this image, and the traced filaments were saved to the output file. An example of such an output file and the Python script used to analyze the results is given in the Supporting Information. It includes all parameters used in the SOAX algorithm to recreate the results published here. This output file is then read by a Python script, which performs the principal component analysis used to determine the rotational angles of the filaments. The results of these steps are further given as png images, and they can be created by running the python script on the SOAX output file.

## Supporting Information Available

Additional images of the experiments performed (PDF)
FilamentSimulation.zip: minimal working example to simulate the filament distribution in an arbitrary shape (ZIP)
SoaxAnalysis.zip: minimal working example how the SOAX output files were analyzed (ZIP)
