# Supplementary material for: Extracellular Cues Govern Shape and Cytoskeletal Organization in Giant Unilamellar Lipid Vesicles
Source: ACS Synth Biol. 2023 Jan 18;12(2):369–74. doi: 10.1021/acssynbio.2c00516 (PMC9942188; doi:10.1021/acssynbio.2c00516)
Supplement: Supplementary file 1 — sb2c00516_si_001.pdf [file sb2c00516_si_001.pdf]

Supplemental Info: Extracellular cues govern shape and cytoskeletal organization in giant unilamellar lipid vesicles

Andreas Fink,[†] Charlotte R. Doll,[†] Ana Yagüe Relimpio,[†,‡] Yannik Dreher,[¶,§] Joachim P. Spatz,[†] Kerstin Göpfrich,[¶,§] and Elisabetta Ada Cavalcanti-Adam[*,†]

†*Department of Cellular Biophysics, Max Planck Institute for Medical Research, Jahnstraße 29, 69120 Heidelberg, Germany*

‡*Institute for Molecular Systems Engineering, University of Heidelberg, Im Neuenheimer Feld 253, 69120 Heidelberg, Germany*

¶*Biophysical Engineering Group, Max Planck Institute for Medical Research, Jahnstraße 29, 69120 Heidelberg, Germany*

§*Department of Physics and Astronomy, Heidelberg University, 69120 Heidelberg, Germany*

E-mail: eacavalcanti@mr.mpg.de

Linewidth of micropatterns

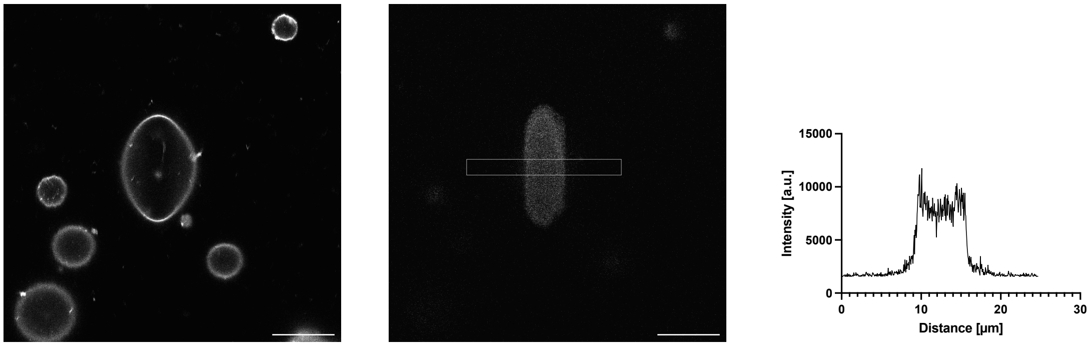

(a) (b) (c)

Figure S1: An example image of an adherent GUV and the respective pattern. The pattern intensity across the line has been plotted. It is evident, that the real pattern is wider than the 2 µm used to create the mask. The actual pattern width in this case is around 6 µm, as it can be seen in the plot. In the following example images of adherent GUVs (see fig S2 - S6) the variability in the pattern width can be seen.

Representative images for all groups shown in Figure 2

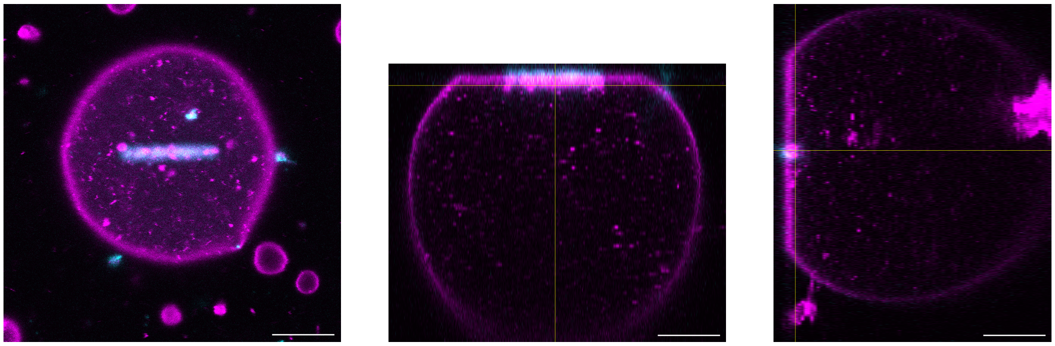

(a) XY-projection (b) XZ-projection (c) YZ-projection

Figure S2: A representative image of a GUV of the group <25 µm is shown. The pattern (cyan) in the XY-projection (a) is shown at the surface, the membrane (magenta) 2 µm above the surface. The scalebar is 10 µm.

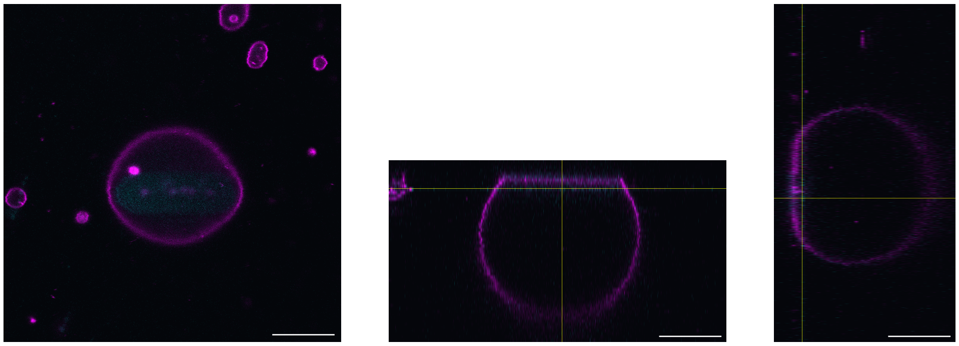

(a) XY-projection (b) XZ-projection (c) YZ-projection

Figure S3: A representative image of a GUV of the group 20-25 µm is shown. The pattern (cyan) in the XY-projection is shown at the surface, the membrane (magenta) 2 µm above the surface. The scalebar is 10 µm.

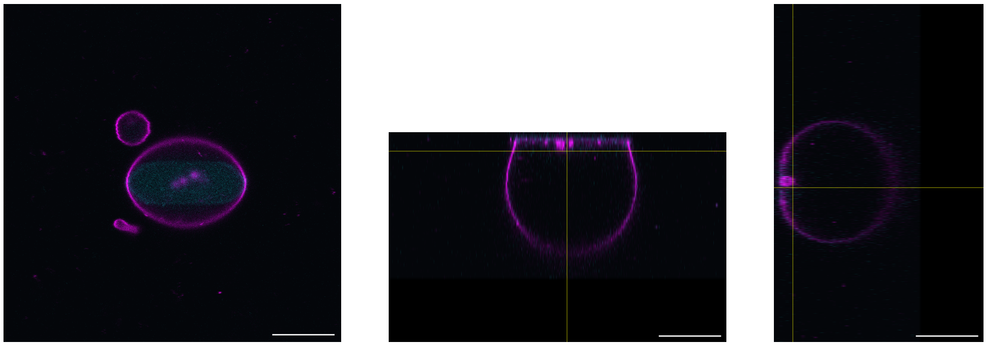

(a) XY-projection (b) XZ-projection (c) YZ-projection

Figure S4: A representative image of a GUV of the group 15-20 µm is shown. The pattern (cyan) in the XY-projection is shown at the surface, the membrane (magenta) 2 µm above the surface. The scalebar is 10 µm.

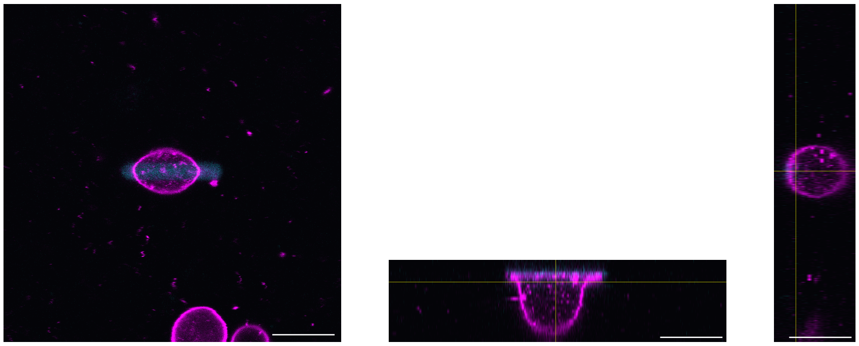

(a) XY-projection (b) XZ-projection (c) YZ-projection

Figure S5: A representative image of a GUV of the group 10-15 µm is shown. The pattern (cyan) in the XY-projection is shown at the surface, the membrane (magenta) 2 µm above the surface. The scalebar is 10 µm.

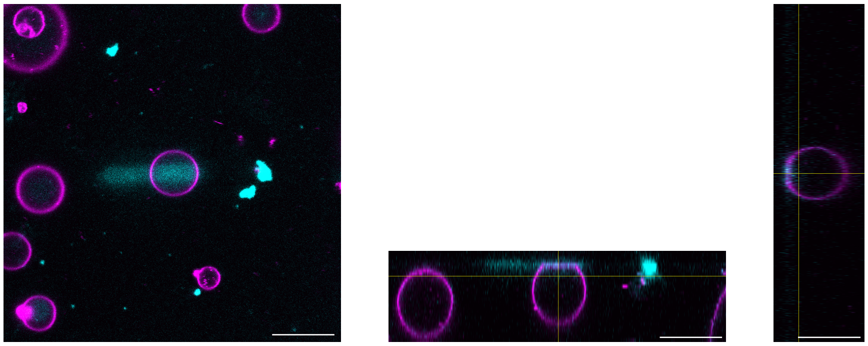

(a) XY-projection (b) XZ-projection (c) YZ-projection

Figure S6: A representative image of a GUV of the group 5-10 µm is shown. The pattern (cyan) in the XY-projection is shown at the surface, the membrane (magenta) 2 µm above the surface. The scalebar is 10 µm.

Actin orientation in GUVs adherent on 10 µm patterns

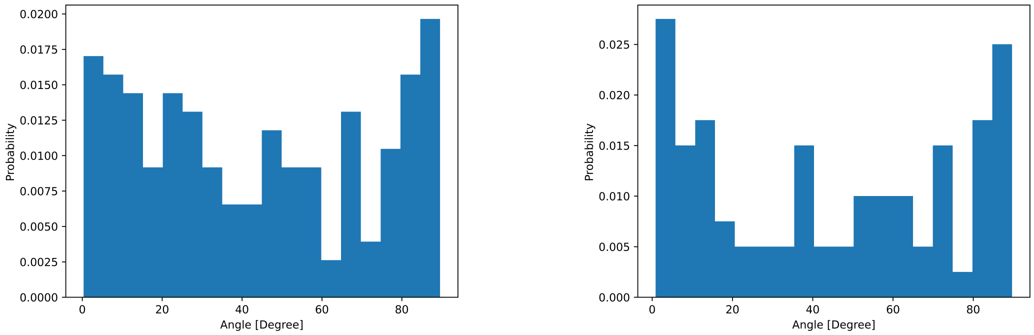

(a) 10 µm cross pattern (b) 10 µm crossbow pattern

Figure S7: Angular distributions of actin filaments in adherent GUVs on 10 µm pattern. A slight asymmetry in the distributions can be seen for both patterns. The simple model presented in figure 4 seems to be able to describe the filament distribution of the cross pattern well. It fails however to describe the asymmetric nature of the crossbow pattern. This can be accounted to the fact that our model assumed actin to be straight rods, which is not expected to hold true for a GUV size significantly smaller than the persistence length. For the cross pattern 7 GUVs from 2 independent experiments (154 filaments) were analyzed. For the crossbow pattern 6 GUVs from 2 independent experiments (81 filaments) were analyzed.

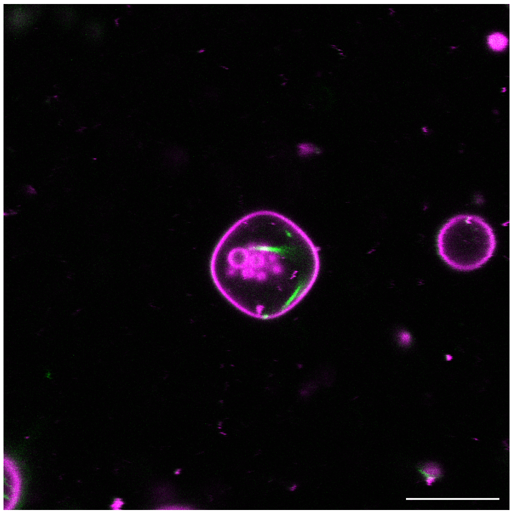

(a) GUV on a 10 µm cross pattern

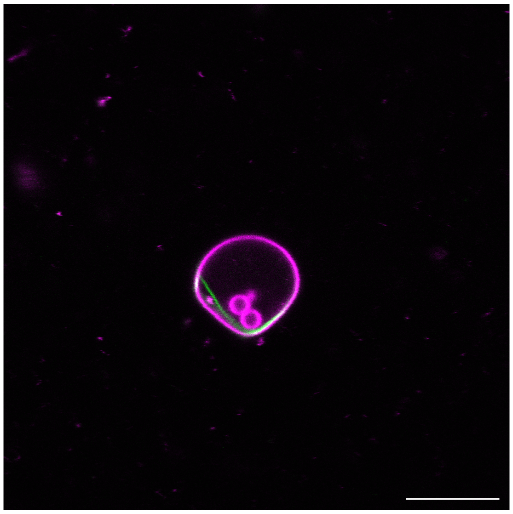

(b) GUV on a 10 µm crossbow pattern

Figure S8: Representative confocal images of GUVs used to create the angular distributions in figure S7. The scale bar is 10 µm. Actin is shown in green, the membrane in magenta. It can be seen, especially in the case of the GUV adhered to the crossbow shape, that a single filament bends along the lower part of the GUV. This behavior is not accounted for in our simple model, and could therefore lead to the failure to describe such a system, where the persistence length of the filament is larger than the size of the compartment.

Confocal images of adherent GUVs on micropatterned surfaces

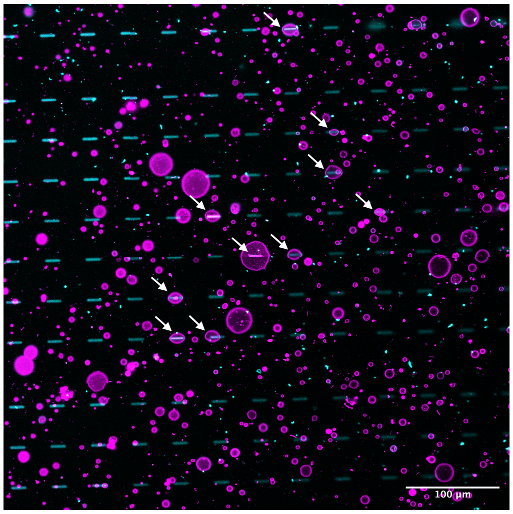

(a) Empty GUVs on 15 µm line pattern.

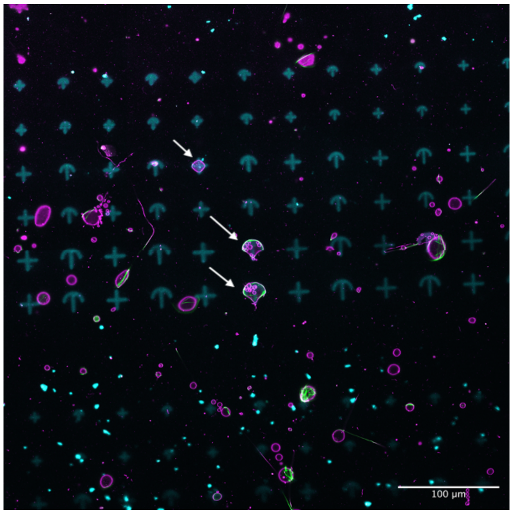

(b) Actin filled GUVs on micropattern

Figure S9: Representative confocal images of GUVs adhering to a micropatterned surface (marked with an arrow). The actin filled GUVs were seeded on cross and crossbow pattern ranging from 10 to 20 µm. It can be seen that the yield is significantly higher with empty GUVS. Further, it should be noted that not all GUVs have the same amount of actin encapsulated. The scale bar is 100 µm. The pattern is shown in cyan, the membrane in magenta and actin, stained with SIR-Actin, in green.
