# Supplementary figures and images for: Extracellular Cues Govern Shape and Cytoskeletal Organization in Giant Unilamellar Lipid Vesicles
Source: ACS Synth Biol. 2023 Jan 18;12(2):369–74. doi: 10.1021/acssynbio.2c00516 (PMC9942188; doi:10.1021/acssynbio.2c00516)

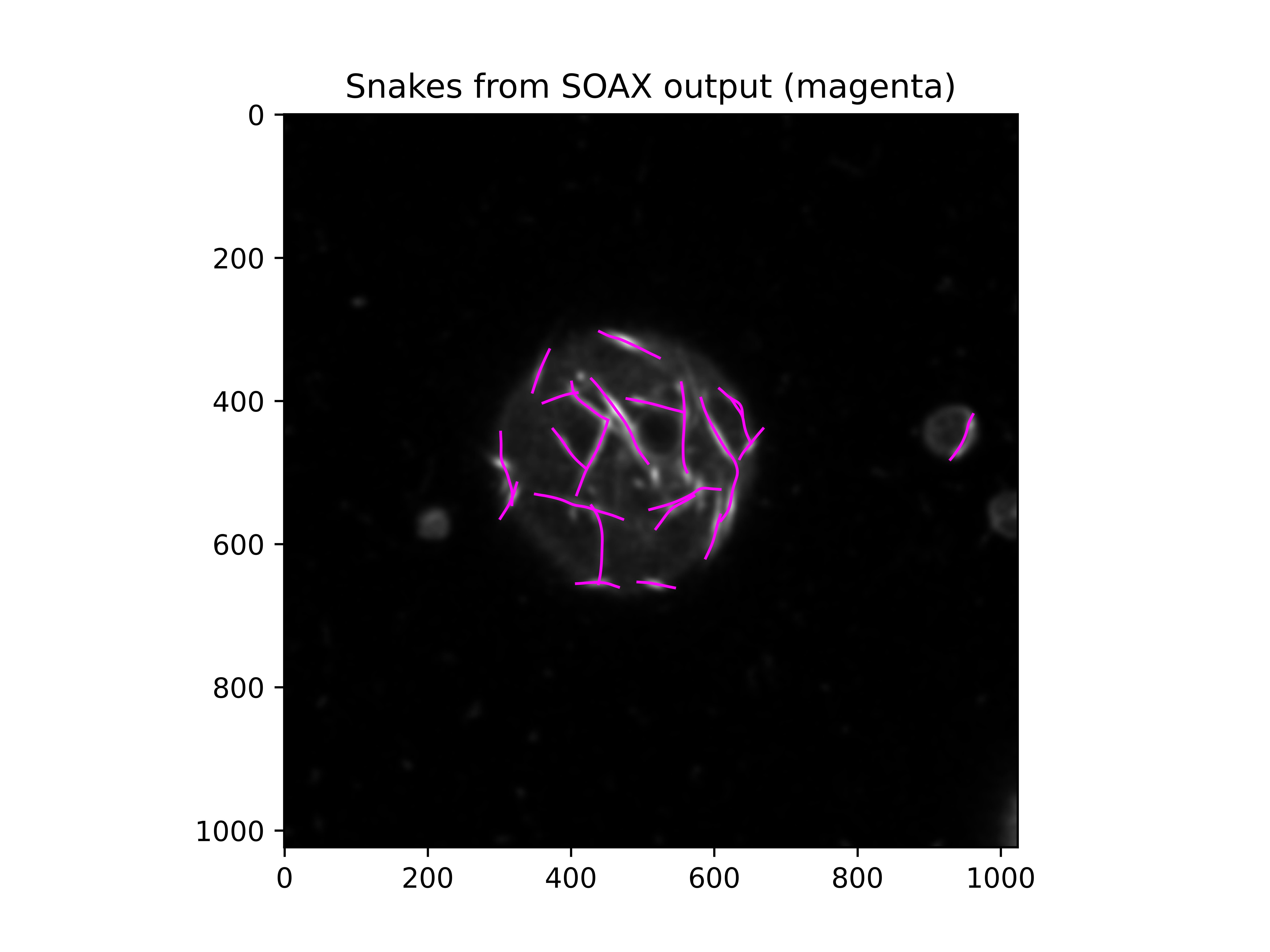

Supplement: Supplementary file 2 — sb2c00516_si_002.zip [file sb2c00516_si_002.zip › SoaxAnalysis/example_guv_after_soax.png]

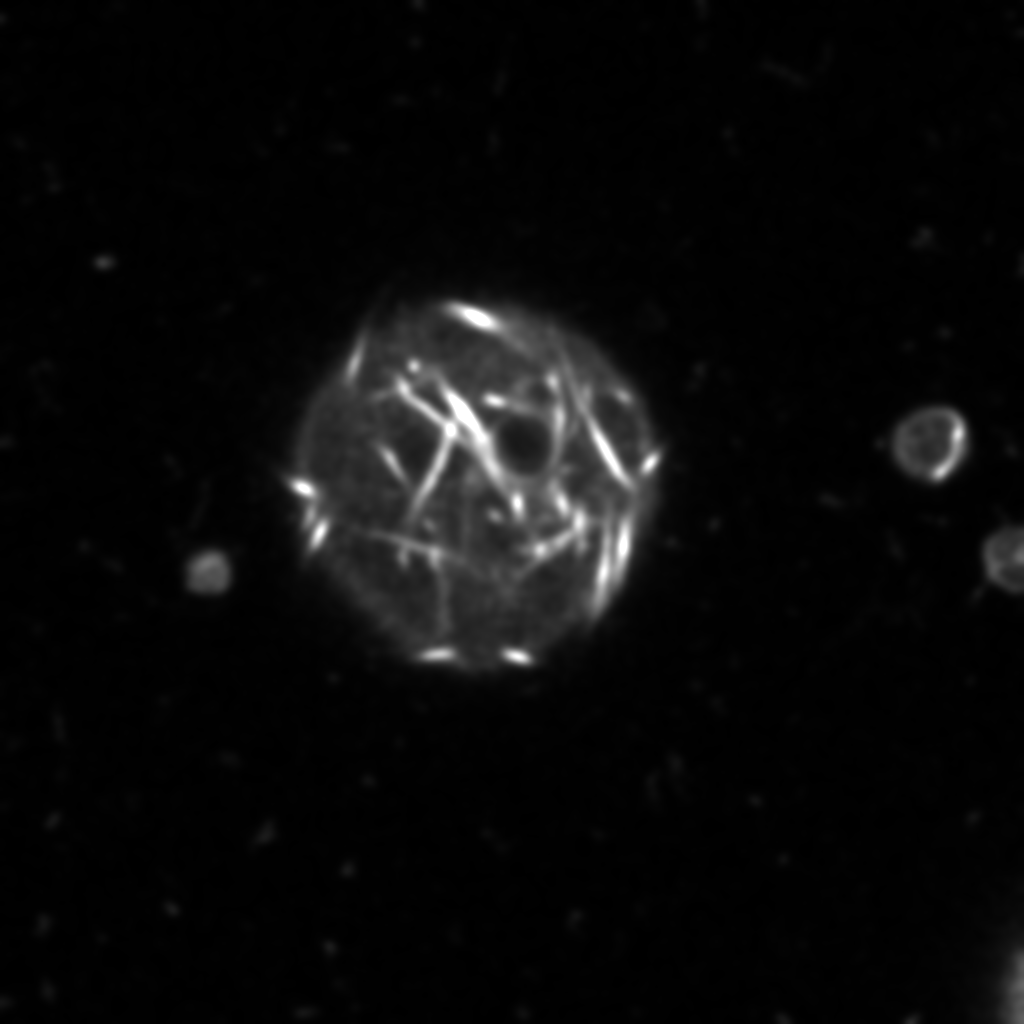

Supplement: Supplementary file 2 — sb2c00516_si_002.zip [file sb2c00516_si_002.zip › SoaxAnalysis/GUV_actin_with_gaussian_blur.tif]

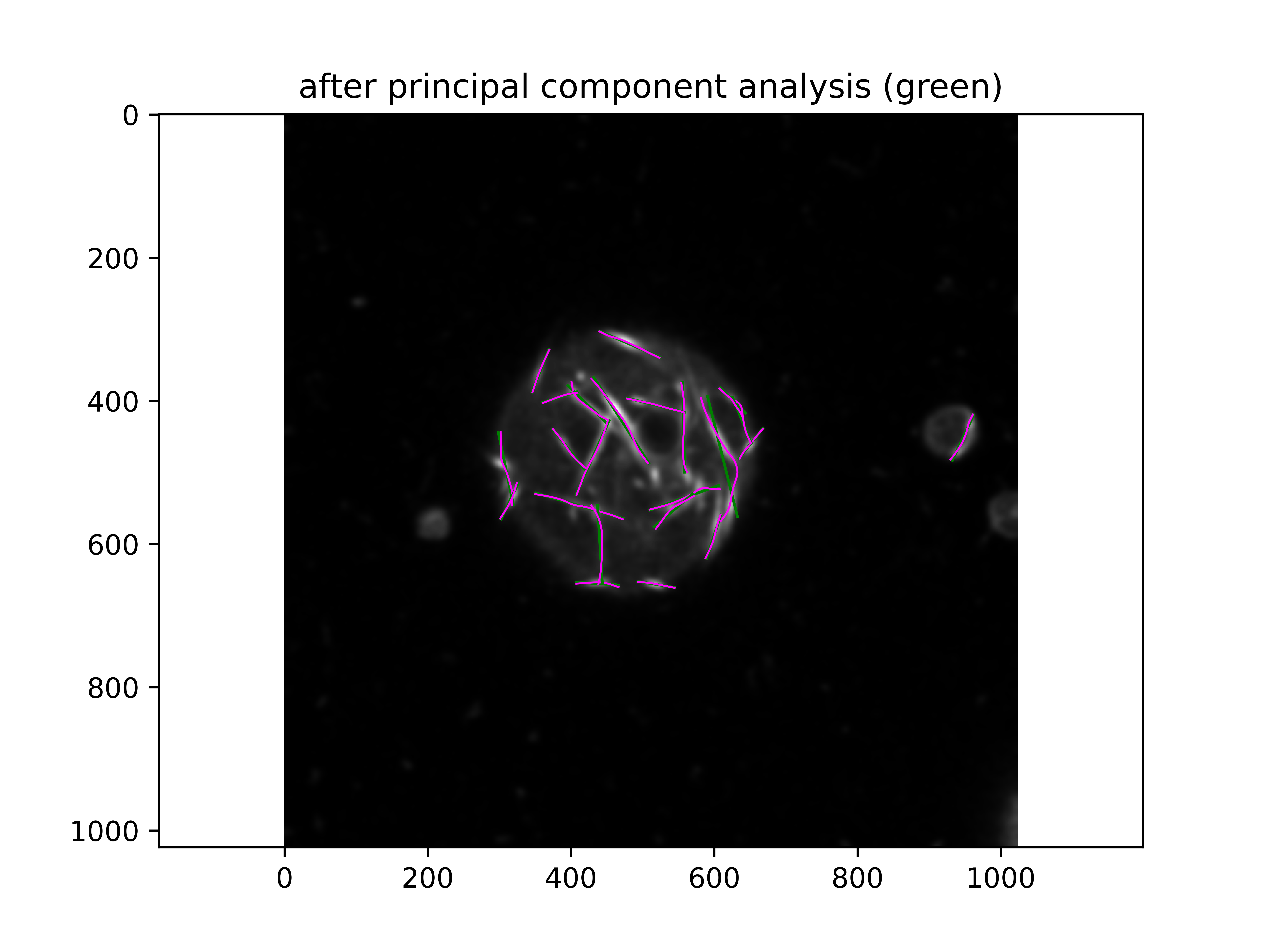

Supplement: Supplementary file 2 — sb2c00516_si_002.zip [file sb2c00516_si_002.zip › SoaxAnalysis/example_guv_after_PCA.png]

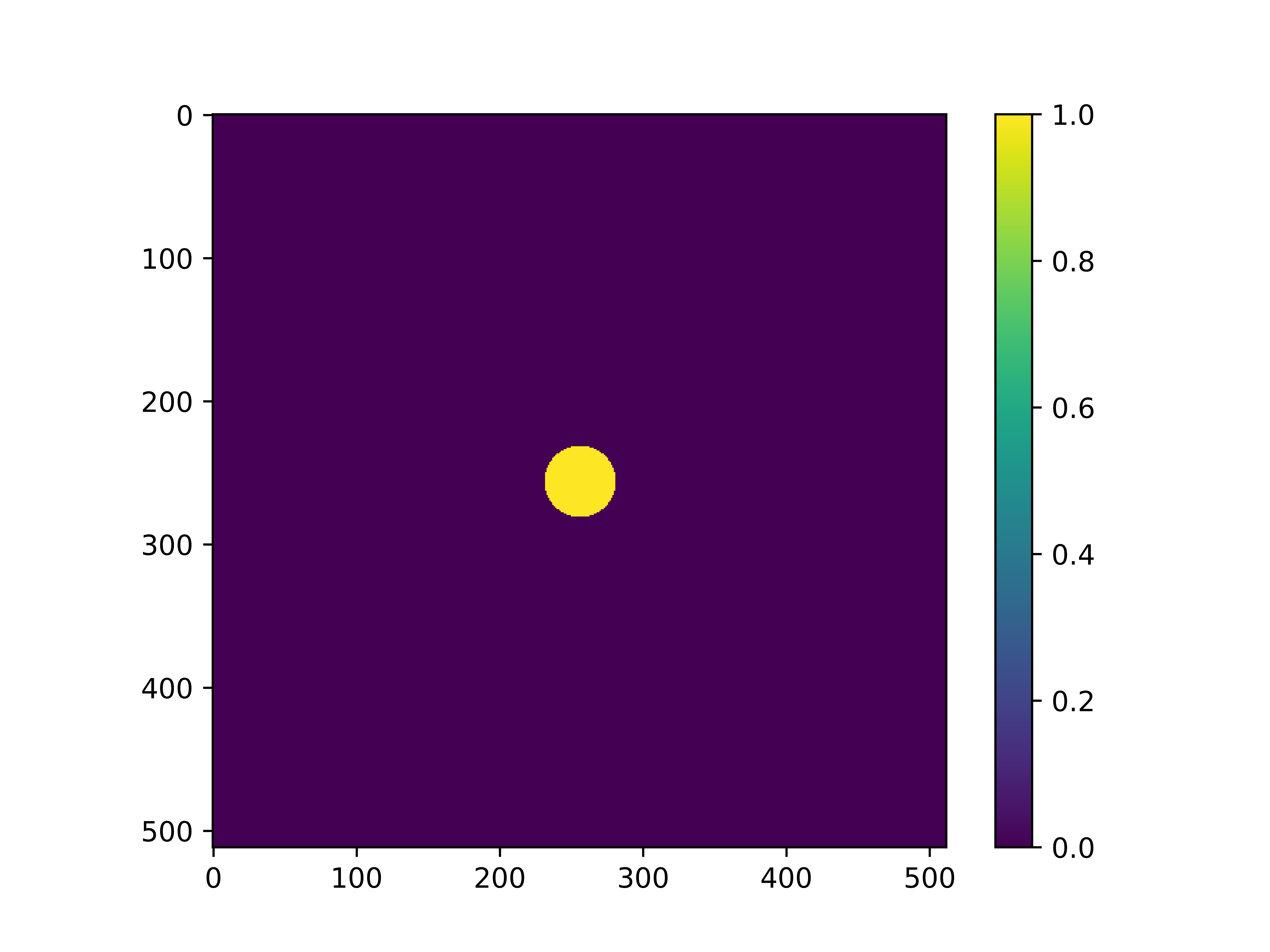

Supplement: Supplementary file 3 — sb2c00516_si_003.zip [file sb2c00516_si_003.zip › FilamentSimulation/binary_image_circle.png]

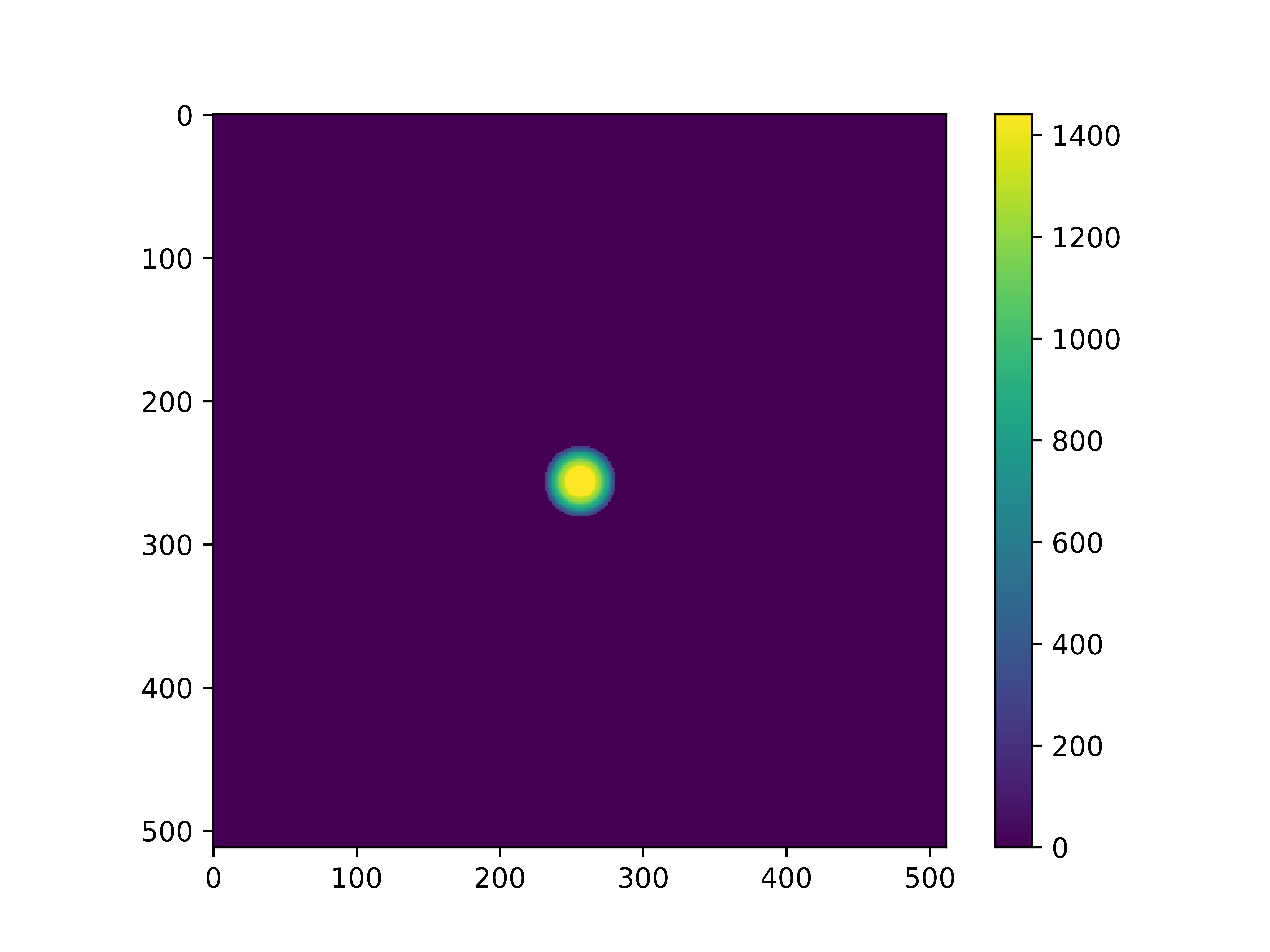

Supplement: Supplementary file 3 — sb2c00516_si_003.zip [file sb2c00516_si_003.zip › FilamentSimulation/simulation_result_image_circle.png]
